# Role of perfusion parameters on DCE-MRI and ADC values on DWMRI for invasive ductal carcinoma at 3.0 Tesla

**DOI:** 10.1186/s12957-018-1538-8

**Published:** 2018-12-21

**Authors:** Fei Liu, Mei Wang, Haige Li

**Affiliations:** Department of Medical Imaging, The Second Affiliated Hospital of Nanjing Medical University, No.121 Jiangjiayuan, Nanjing, 210011 Jiangsu Province China

**Keywords:** Diffusion-weighted MRI, Magnetic resonance imaging, Invasive ductal carcinoma, Breast cancer

## Abstract

**Background:**

The value of apparent diffusion coefficient (ADC) values and quantitative parameters (Ktrans, Kep, Ve) in detecting prognostic factor at 3.0 Tesla remains unclear, especially in predicting prognosis of breast cancer.

**Methods:**

A total of 151 patients with IDC underwent breast DCE-MRI and DWI-MRI at 3.0 Tesla following surgery. The ADC values were acquired with *b* values of 0 and 1000 s/mm^2^. The relationship between ADC values or DCE-MRI quantitative parameters and size, histologic grade (HG), lymph node metastasis (LNM), ER, PR, and Ki67 was evaluated. The predictive values of ADC, Ktrans, Kep, and Ve to prognosis of IDC were assessed.

**Results:**

ADC value was positively related to size (*P* = 0.04) and HER2 (*P* = 0.046) expression and negatively related to ER (*P* = 0.012) and PR (*P* < 0.001) expression. Ktrans value has positive correlation with size (*P* < 0.001), HG (*P* < 0.001), LNM (*P* < 0.001), HER2 (*P* = 0.007), and Ki67 (*P* < 0.001) expression and negative correlation with ER (*P* < 0.001) and PR (*P* < 0.001) expression. Kep value was positively related to size (*P* < 0.001) and negatively related to ER (*P* < 0.001) and PR (*P* < 0.001) expression. Ve value was negatively related to HER2 expression (*P* = 0.004). The Cox hazard ratio (HR) of ADC, Ktrans, Kep, and Ve values on survival was 5.26 (*P* = 0.093), 1.081 (*P* = 0.002), 1.006 (*P* = 0.941), and 0.883 (*P* = 0.926), respectively.

**Conclusions:**

Ktrans value was a best predictive indicator of HG, LNM, ER, PR, and Ki67 expression, and ADC value was the best predictive indicator of HER2. Preoperative use of the 3.0 Tesla could provide important information to determine the optimal treatment plan.

## Background

Breast cancer is a highly heterogeneous disease with multiple biological subtypes that are distinguished by different clinical, pathological, and molecular characteristics, treatment measures, and prognosis [1]. The morphological and cytological features of the tumor are closely related to the degree of malignancy of the tumor. Multi-gene expression analysis demonstrated that this clinical feature of breast cancer is a result of potential gene expression or inhibition [2]. Currently recognized cytokines that evaluate the therapeutic effect and prognosis of breast cancer include estrogen receptor (ER), progesterone (PR), human epidermal growth factor receptor (2), human epidermal growth factor receptor-2 (HER-2), and nucleus-associated antigen Ki-67, which play a synergistic role in the development of breast cancer via influencing the occurrence, development, and prognosis of breast cancer [3, 4].

Early diagnosis of breast cancer plays an important role in the choice of treatment regimen and the prognosis of the disease [5]. Dynamic contrast-enhanced magnetic resonance imaging (DCE-MRl) is a noninvasive diagnosis of assessing the internal microenvironment of the tumor in recent years [6]. The imaging technique can reflect the characteristics of blood flow, microvascular permeability, vascular density, tissue oxygen concentration, and even tissue metabolic level [6, 7]. Studies had confirmed that DCE-MRI can evaluate and predict the clinical efficacy and prognosis of bladder cancer, bone tumor, and breast cancer [8, 9].

Currently, magnetic resonance imaging has become one of the indispensable means of examination for breast disease and breast-conserving surgery [10]. In addition, magnetic resonance imaging can also be used to assess tumor functional changes through special examinations such as diffusion-weighted imaging (DWI) and hydrogen proton resonance spectroscopy (1H-MRS) [11, 12]. Furthermore, it is important to evaluate the number of tumors, the extent of invasion of lesions, and lymph node metastasis (LNM) and TNM staging in patients with neoadjuvant chemotherapy and post-chemotherapy evaluation [13, 14]. In particular, DWI is capable of imaging the microscopic diffusion of water molecules, which can identify the function and metabolic state of tumor tissues and quantify the dispersion of water molecules through ADC values [15].

The previous investigations had reported the average values of ADC in malignant and benign breast tumors and the threshold values of ADC that distinguished malignancies from benign lesions [15–17]. However, there only were few reports of applications of both DWI and DCE-MRl on the invasive ductal carcinoma (IDC) evaluation. The aim of this study was to assess the correlations between parameters of DWI and DCE-MRl in IDC of the breast and prognosis or prognostic factors, such as tumor size, histologic grade, lymph nodes metastasis (LNM), estrogen receptor (ER), progesterone receptor (PR), HER2 (c-erb-2), and Ki-67.

## Methods

This is a prospective study that was approved by our hospital institutional review board, and informed consent was obtained from all patients.

A total of 207 patients with breast cancer who were admitted to our hospital from March 2010 to April 2012 were all female and aged from 31 to 77 years with an average of 52 ± 10.3 years. Among them, 151 patients who were diagnosed with histopathological IDC of the breast by pathological diagnosis postoperatively were enrolled in this study. There were 56 patients who had final diagnoses of other breast cancers, including ductal carcinoma in situ (DCIS) (*n* = 28), invasive lobular carcinoma (*n* = 5), intraductal papillary carcinoma (*n* = 4), and DCIS with microinvasion (*n* = 19), excluded. The average age of the 151 patients enrolled in this study was 51.7 ± 10.6. In addition to surgical treatment, information on other treatment methods for patients with IDC, including surgical methods, chemotherapy, and endocrine therapy, was collected.

### Image acquisition

The breast MRI studies were performed on a MAGNETOM Verio 3.0 Tesla (T) scanner (Siemens Medical Solutions, Germany) using a dedicated 16-channel breast-specific phased array surface coil with patients in the prone position. Parameters for several sequences were listed as follows: T2-weighted imaging (T2WI): TR/TE, 4000/70 ms; slice thickness, 5 mm, 0.5 mm gap; field of view (FOV), 340 × 340; matrix, 448 × 448; NEX,1. T1WI (FLASH-3D): TR/TE, 3.6/2.1 ms; slice thickness, 1.2 mm, 0.2 mm gap; FOV, 340 × 340; matrix, 448 × 380; flip angle, 10°; NEX,0.7. EPI-DWI: TR/TE, 8300/85 ms; slice thickness, 4 mm, 2 mm gap; FOV, 360 × 147; matrix, 220 × 220; NEX,3; performed by fat suppression technology with *b* values of 0 and 1000 s/mm^2^. DCE-MRI was performed by the following parameters: TR/TE, 4.5/1.6 ms; flip angle, 10°; slice thickness,1 mm, 0.2 mm gap; matrix, 448 × 344; FOV, 340 × 340; NEX, 1. The first phase scan was conducted before the contrast agent was injected. After 25 s, Gd-DTPA (0.1–0.15 mmol/kg) was injected intravenously at a flow rate of 3.0 ml/s with a high-pressure syringe, followed by administration of a saline flush. The acquisition time was 487 s (77 s per dynamic sequence).

#### Images interpretation

All images were processed on a Siemens post-processing workstation. Three imaging diagnosticians were randomly assigned to read and analyze the MRI information of patients, and they were blinded from histological results. The inconsistencies in the results are agreed upon by consensus.

The quantitative DCE-MRI parameters were calculated by non-linear least squares method using the two-compartment model of Tofts et al. in the German Siemens MRI Workstation 4D tissue software [18]. Quantitative DCE-MRI parameters include volume transfer constant (Ktrans), rate constant (Kep), and extravascular space (Ve). The maximum diameter of the lesion was selected as the region of interest (ROI), and the average value was obtained from three times determinations with avoiding the liquefaction, necrosis, and bleeding area of tumor tissue and other features. The size of the tumor and the enhanced morphology were obtained by enhancing images. According to the different enhanced morphologies of breast cancer, tumors were divided into enhanced lump group and non-lump enhanced group.

By using Mean Curve software from Siemens post-processing workstation, the lesion with higher signal intensity was selected as the region of interest (ROI) to draw time-signal intensity curve (TIC). Two different areas of the lesion were selected, and three different ROIs were taken for each area. The ROI sizes ranged from 15.0 to 420.6 mm^2^.

For measurements of ADC values, three ROIs were selected within the main lesions and mean values were calculated with *B* = 0, 1000 s/mm^2^ as the apparent diffusion coefficient of the *b* value. All measurements were performed independently by two mammography diagnostic specialists with at least 4 years of experience.

### Histological analysis

Histological grading of IDC was performed based on nuclear polymorphic tubular structures and mitotic counts according to the modified criteria of Bloom and Richardson [17]. Historical analysis was performed by surgically resected specimens. A score of 3–5, 6–7, and 8–9 were considered grade I, grade II, and grade III, respectively. According to the International Union Against Cancer (UICC) TNM staging system, breast cancer size was divided into three groups: maximum diameter of the tumor < 2 cm (size 1), 2 cm–5 cm (size 2), and ≥ 5 cm (size 3). The lymph node metastasis was assessed by routine staining. Immunohistochemistry of ER, PR, Ki-67, and HER2 (c-erbB-2) was analyzed as molecular markers. By routine staining procedures, the case with more than 10% of cancer cells with nuclear staining was regarded as ER, PR, or Ki-67 positivity. Scores of 0 (negative), were considered negative for expression of proteins, scores of 1+ and 2+ (moderate) were regarded as positive for expression of proteins, and scores of 3+ (strong) was considered positive for over-expression of proteins.

### Follow-up

Follow-up is performed every 2 months for 60 months. The overall survival is defined as the time from diagnosis of any cause to death.

### Statistical analysis

SPSS 22 software was used for statistical analysis. Data for tumor size, histologic grade, ER, PR, Ki-67, and HER2 were defined as group 1, group 2, and group 3 according to their classifications, respectively (Table 1). The data was assessed by test of normality, and the data corresponding to the normal distribution were expressed as *x* ± *s*. The data of non-normal distribution were expressed as the median. Spearman correlation test was conducted to calculate the relationships between ADC values or DCE-MRI quantitative parameters and prognostic factors. *P* < 0.05 was considered statistically significant. One-way ANOVA test (analysis of variance) was performed to test the significance of differences between three or more groups. Cox regression model was conducted to evaluate the prognostic value of ADC values or DCE-MRI quantitative parameters.

**Table 1:** General information of patients (*n* = 151)

Prognostic factors	Group	No. of cases	ADC	Ktrans	Kep	Ve
Size						
< 2 cm	1	55	1.04 ± 0.23	12.00 ± 0.65	0.64 ± 1.41	0.19 ± 0.28
2–5 cm	2	80	1.16 ± 0.25	18.15 ± 8.50	2.90 ± 6.59	0.20 ± 0.37
> 5 cm	3	16	1.09 ± 0.15	15.98 ± 8.12	1.58 ± 1.63	0.09 ± 0.12
TNM stage						
T1/2	1	112	1.10 ± 0.24	13.96 ± 7.32	1.73 ± 4.51	0.18 ± 0.29
T3/4	2	39	1.13 ± 0.23	21.78 ± 7.57	2.52 ± 6.24	0.18 ± 0.39
HG score						
3–5	1	19	1.00 ± 0.27	7.73 ± 4.89	2.99 ± 3.76	0.28 ± 0.34
6–7	2	72	1.15 ± 0.24	15.21 ± 7.99	1.47 ± 4.60	0.14 ± 0.27
8–9	3	60	1.10 ± 0.23	19.51 ± 6.94	2.16 ± 5.76	0.21 ± 0.36
LNM						
Positive	1	83	1.09 ± 0.28	13.96 ± 8.30	2.61 ± 6.37	0.24 ± 0.39
Negative	2	68	1.13 ± 0.18	18.43 ± 7.22	1.11 ± 2.26	0.11 ± 0.17
ER expression						
Negative	1	60	1.15 ± 0.25	19.02 ± 6.48	2.00 ± 5.77	0.20 ± 0.36
Moderate	2	36	1.12 ± 0.29	16.76 ± 10.71	0.97 ± 1.76	0.13 ± 0.26
Strong	3	55	1.06 ± 0.18	12.15 ± 6.11	2.49 ± 5.50	0.21 ± 0.31
PR expression						
Negative	1	68	1.16 ± 0.28	19.22 ± 6.85	2.98 ± 6.92	0.21 ± 0.38
Moderate	2	48	1.10 ± 0.23	13.31 ± 9.04	1.83 ± 2.73	0.25 ± 0.31
Strong	3	35	1.02 ± 0.12	13.33 ± 6.86	0.05 ± 0.12	0.05 ± 0.10
HER2 expression						
Negative	1	64	1.02 ± 0.24	14.28 ± 8.94	3.91 ± 7.17	0.33 ± 0.42
Moderate	2	59	1.12 ± 0.18	16.60 ± 7.19	0.53 ± 1.09	0.06 ± 0.12
Strong	3	28	1.29 ± 0.27	18.55 ± 7.35	0.40 ± 0.78	0.11 ± 0.19
Ki67 expression						
Negative	1	19	1.12 ± 0.15	8.60 ± 4.80	6.18 ± 8.19	0.29 ± 0.36
Moderate	2	92	1.09 ± 0.25	15.49 ± 6.75	0.63 ± 1.30	0.13 ± 0.24
Strong	3	40	1.15 ± 0.26	20.61 ± 9.36	2.92 ± 6.90	0.26 ± 0.42
SM						
MRM	1	130	1.10 ± 0.22	16.89 ± 8.12	1.93 ± 5.22	0.19 ± 0.33
BCS	2	21	1.17 ± 0.33	10.31 ± 5.45	1.96 ± 3.48	0.16 ± 0.26
AC						
Yes	1	139	1.04 ± 0.28	9.59 ± 5.52	0.96 ± 1.81	0.18 ± 0.31
No	0	12	1.12 ± 0.24	16.58 ± 8.07	2.03 ± 5.20	0.18 ± 0.32
RT						
Yes	1	88	1.08 ± 0.25	12.31 ± 7.41	2.34 ± 5.25	0.18 ± 0.31
No	0	63	1.13 ± 0.24	18.61 ± 7.60	1.65 ± 4.83	0.19 ± 0.33
ET						
Yes	1	92	1.09 ± 0.29	11.76 ± 7.03	2.25 ± 4.84	0.20 ± 0.30
No	0	59	1.12 ± 0.20	18.68 ± 7.63	1.74 ± 5.12	0.18 ± 0.33

*HG* histologic grade, *LNM* lymph node metastasis, *SM* surgical methods, *MRM* modified radical mastectomy, *BCS* breast-conserving surgery, *AC* adjuvant chemotherapy, *RT* radiation therapy, *ET* endocrine therapy

## Results

### The correlations between prognostic factors and ADC values or DCE-MRI quantitative parameters

The correlations between prognostic factors and ADC values or DCE-MRI quantitative parameters are listed in Table 2. There was a significant relationship between ADC values and size, ER, PR, or HER, and the corresponding correlation coefficients were 0.167 (*P* = 0.041), − 0.163 (*P* = 0.046), − 0.205 (*P* = 0.012), and 0.344 (*P* < 0.0001), respectively. There was a significant correlation between Ktrans values and all prognostic factors, including size, TNM, HG, LNM, ER, PR, HER2, and Ki67, and the corresponding correlation coefficients were 0.039 (*P* < 0.0001), 0.459 (*P* < 0.0001), 0.459 (*P* < 0.0001), 0.285 (*P* < 0.0001), − 0.377 (*P* < 0.0001), − 0.390 (*P* < 0.0001), 0.217 (*P* = 0.007), and 0.390 (*P* < 0.0001), respectively. There was a significant relationship between Kep values and size, TNM, PR, and HER2, and the corresponding correlation coefficients were 0.412 (*P* < 0.0001), 0.194 (0.017), − 0.287 (*P* < 0.0001), and – 0.289 (*P* < 0.0001), respectively. Ve values have significant correlation with only one prognostic factor HER2, and correlation coefficients were − 0.233 (*P* = 0.004).

**Table 2:** The associations between prognostic factors and ADC values or DCE-MRI quantitative parameters

		Size	TNM	HG	LNM	ER	PR	HER2	Ki67
ADC	rho	0.167*	0.087	0.018	0.109	− 0.163*	− 0.205*	0.344**	0.042
	*P*	0.041	0.287	0.827	0.1849	0.046	0.012	< 0.001	0.609
Ktrans	rho	0.390**	0.459**	0.459**	0.285**	− 0.377**	− 0.390**	0.217**	0.390**
	*P*	< 0.001	< 0.001	< 0.001	< 0.001	< 0.001	< 0.001	0.007	< 0.001
Kep	rho	0.412**	0.194*	− 0.061	0.097	0.071	− 0.287**	− 0.289**	− 0.023
	*P*	< 0.001	0.017	0.456	0.237	0.387	< 0.001	< 0.001	0.778
Ve	rho	0.030	− 0.051	− 0.055	− 0.038	0.002	− 0.040	− 0.233**	− 0.021
	*P*	0.712	0.534	0.502	0.639	0.976	0.626	0.004	0.796

*HG* histologic grade, *LNM* lymph node metastasis

*P < 0.05; **P < 0.01

### Comparisons of enhancement quantitative parameters or ADC values with subgroups of prognostic factors

Multiple comparisons of enhancement quantitative parameters or ADC values with subgroups of prognostic factors were performed by one-way ANOVA test, and the results are shown in Table 3. Significant differences of ADC values within subgroups of size (*P* = 0.018), PR (*P* = 0.024), and HER2 (*P* < 0.001) were observed. For Ktrans values, significant differences existed in subgroups of all prognostic factors, including size (*P* < 0.001), TNM (*P* < 0.001), HG (*P* < 0.001), LNM (*P* < 0.001), ER (*P* < 0.001), PR (*P* < 0.001), HER2 (*P* = 0.05), and Ki67 (*P* < 0.001). For Kep values, significant differences were observed in subgroups of size (*P* = 0.033), PR (*P* = 0.018), HER2 (*P* < 0.001), and Ki67 (*P* < 0.001). Significant differences of ADC values existed in subgroups of LNM (*P* = 0.011), PR (*P* = 0.014), HER2 (*P* < 0.001), and Ki67 (*P* = 0.031).

**Table 3:** The *P* value of multiple comparisons of enhancement quantitative parameters or ADC values with subgroups of prognostic factors

Prognostic factors	ADC	Ktrans	Kep	Ve
Size	0.018*	< 0.001**	0.033*	0.432
TNM	0.451	< 0.001**	0.400	0.962
HG	0.068	< 0.001**	0.452	0.140
LNM	0.378	0.001**	0.067	0.011**
ER	0.134	< 0.001**	0.364	0.474
PR	0.024*	< 0.001**	0.018*	0.014*
HER2	< 0.001**	0.05*	< 0.001**	< 0.001**
Ki67	0.394	< 0.001**	< 0.001*	0.031*

*HG* histologic grade, *LNM* lymph node metastasis

*P < 0.05; **P < 0.01

**Fig. 1:**
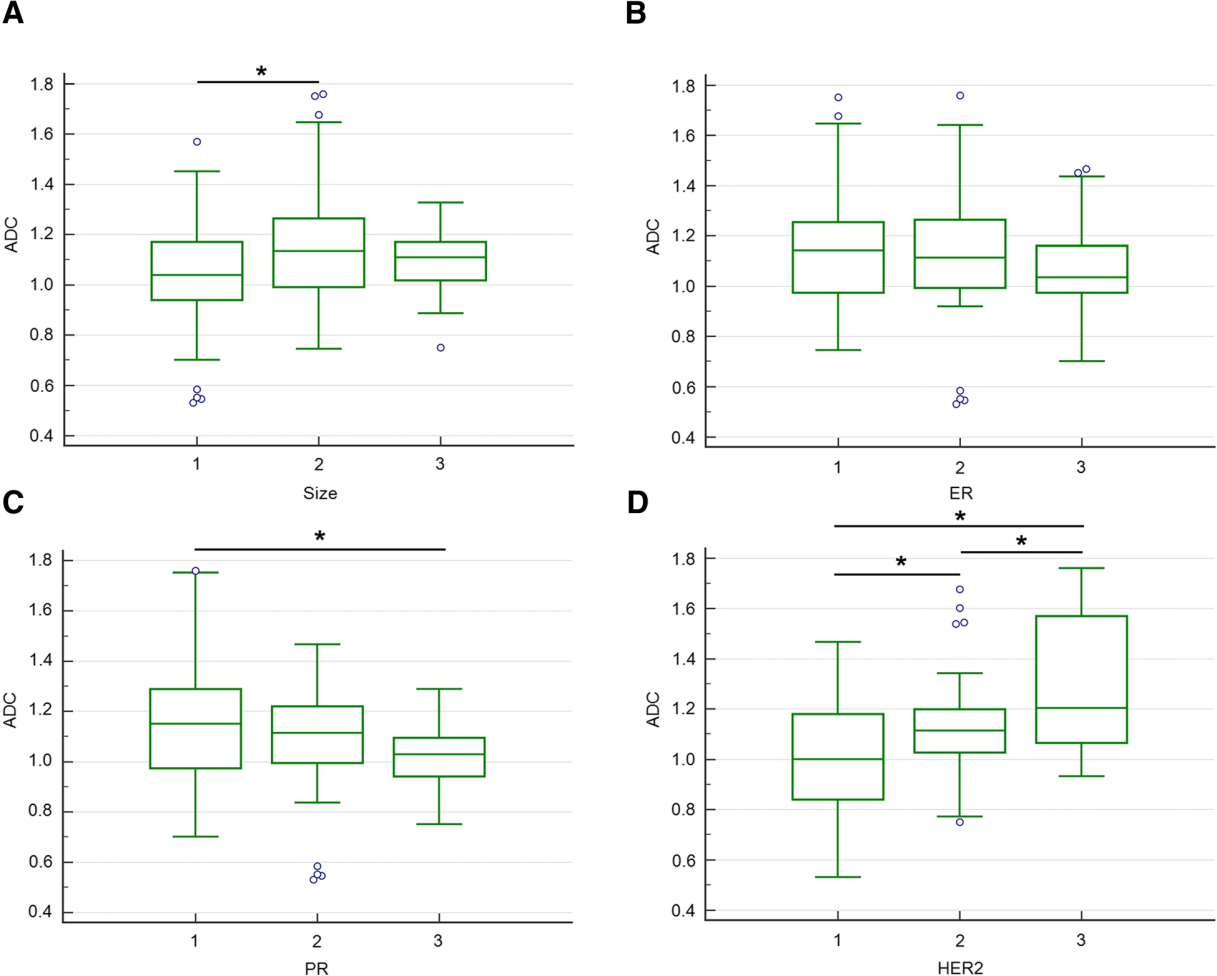
Comparisons of ADC values between subgroups of prognostic factors (**a** size, **b** ER, **c** PR, **d** HER2). Bonferroni post hoc pairwise comparison was used. *P < 0.05

**Fig. 2:**
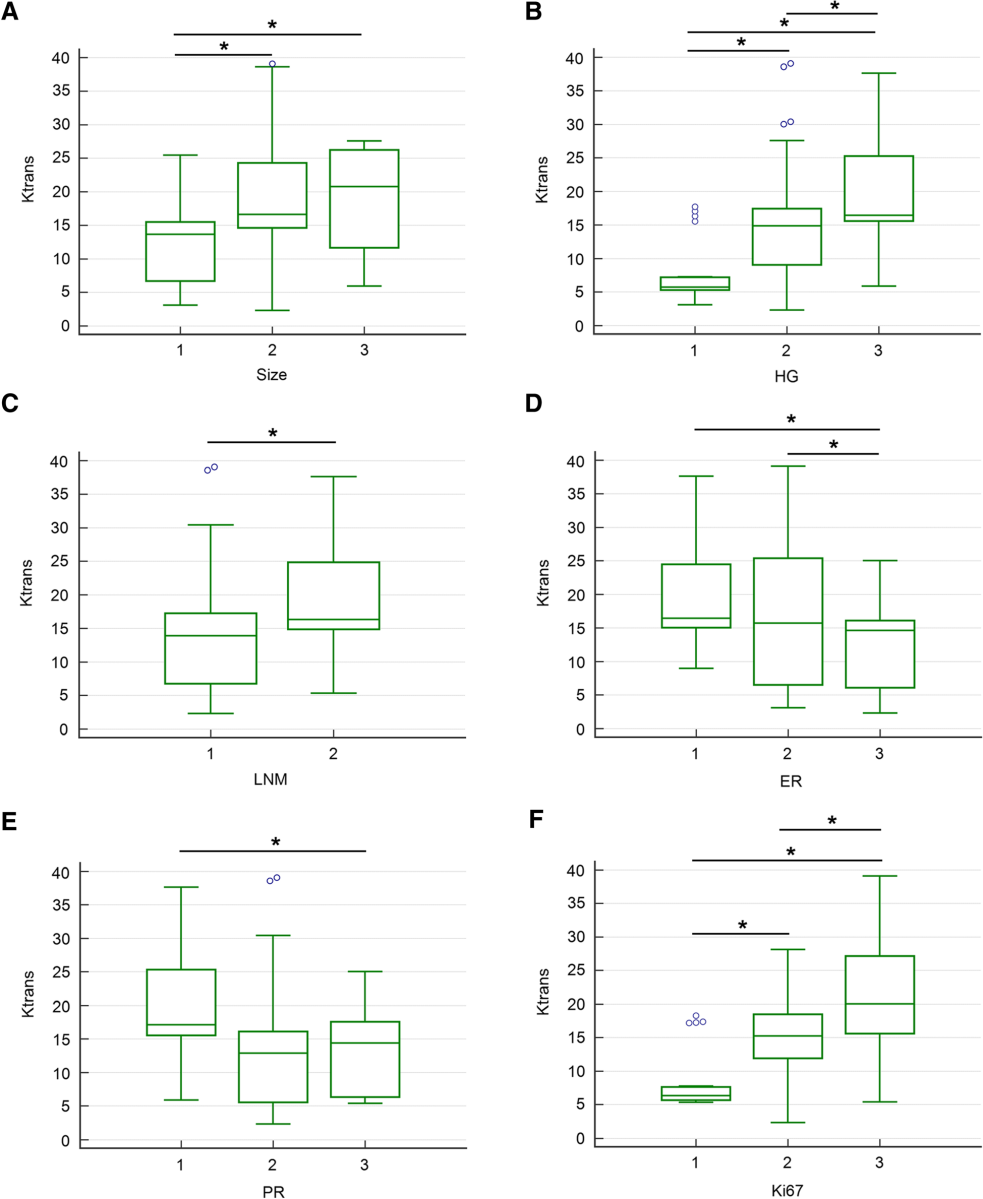
Comparisons of Ktrans values between subgroups of prognostic factors (**a** size, **b** HG, **c** LNM, **d** ER, **e** PR, **f** Ki67). Bonferroni post hoc pairwise comparison was used. HG: histologic grade; LNM: lymph node metastasis; *P < 0.05

**Fig. 3:**
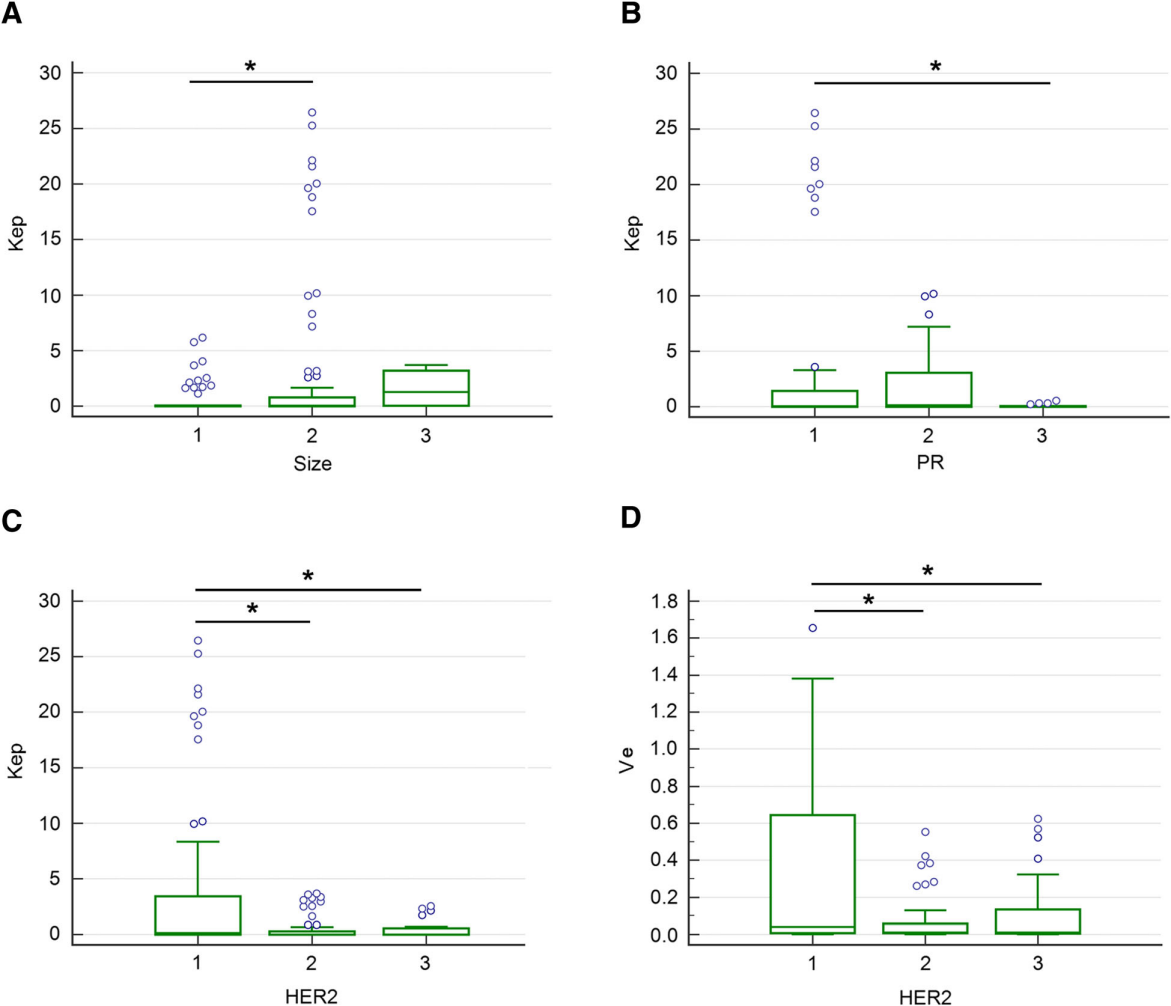
Comparisons of Kep or Ve values between subgroups of prognostic factors (Kep: **a** size, **b** PR, **c** HER2; Ve: **d** HER2). Bonferroni post hoc pairwise comparison was used. *P < 0.05

Bonferroni post hoc pairwise comparisons showed that for tumor size, the ADC values were significantly lower in group 1 than those in group 2, for PR, the ADC values were higher in group 1 than those in group 3, and for HER2, the ADC values were higher in group 3 than those in group 1 or group 2 (Fig. 1). The Ktrans values in size group 2 or 3 were higher than those in group 1. Significant differences of Ktrans values existed in every two groups of HG, and the highest Ktrans value was observed in HG group 3. Higher Ktrans value was showed in LNM group 2 compared with group 1. The Ktrans values were negatively associated with ER expression, and the Ktrans values in group 1 or 2 were significantly higher compared to that in group 3. The Ktrans values were highest in PR group 1, and significant difference of Ktrans values was observed between group 1and group 3. The Ktrans values were positively correlated with Ki67 expression. Higher Ktrans values were observed in higher expression of Ki67 (Fig. 2). The relationships between Kep or Ve values and prognostic factors were complex. However, higher Kep values were observed in size group 2, PR group 1, and HER2 group 1. Similarly, the highest Ve value was observed in HER2 group 1 compared to HER2 group 2 or 3, respectively (Fig. 3).

### Prognostic value of ADC values or DCE-MRI quantitative parameters

**Fig. 4:**
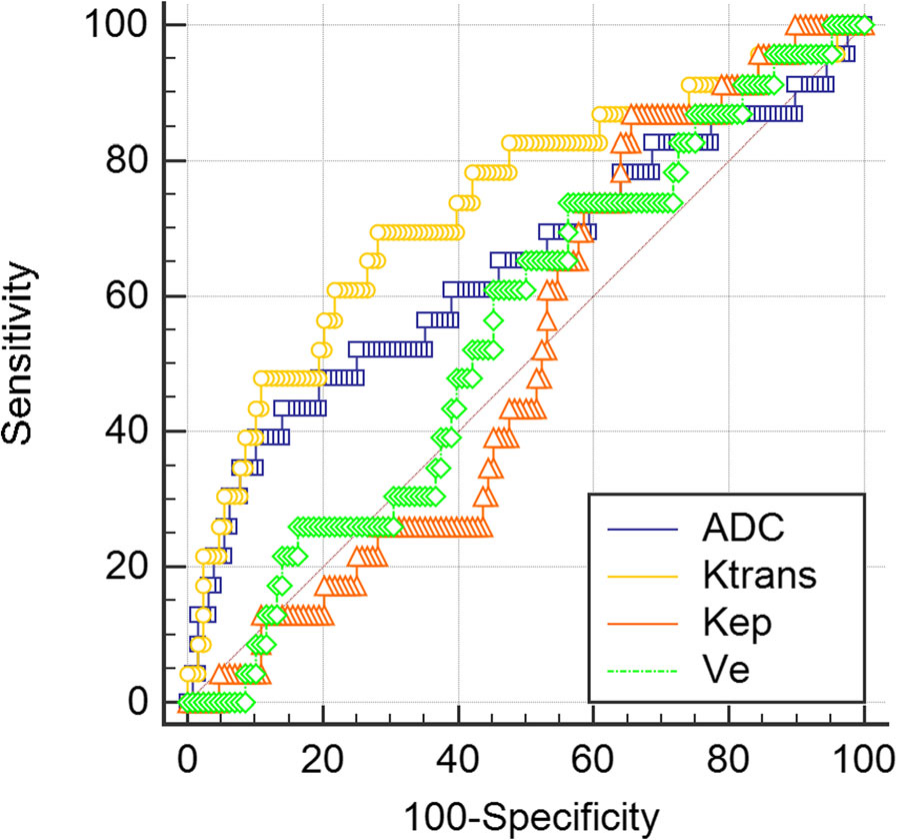
ROC for ADC values or DCE-MRI quantitative parameters versus prognosis of breast cancer

To assess the predictive value of ADC values or DCE-MRI quantitative parameters to the prognosis of breast cancer, ROC curve was conducted (Fig. 4). The AUC of ADC, Ktrans, Kep, and Ve values are listed in Table 4. The highest AUC was achieved by Ktrans values (0.731, 95% CI 0.653 to 0.800) and which was followed by ADC values (0.642, 95% CI 0.560 to 0.718). However, Kep and Ve showed no association with prognosis.

**Table 4:** Area under curve (AUC) of ROC for ADC values or DCE-MRI quantitative parameters versus prognosis of breast cancer

Variable	AUC	SE^a^	95% CI^b^
ADC	0.642	0.0723	0.560 to 0.718
Ktrans	0.731	0.0625	0.653 to 0.800
Kep	0.518	0.0571	0.435 to 0.600
Ve	0.549	0.0616	0.466 to 0.630

^a^DeLong et al., 1988

^b^Binomial exact

**Fig. 5:**
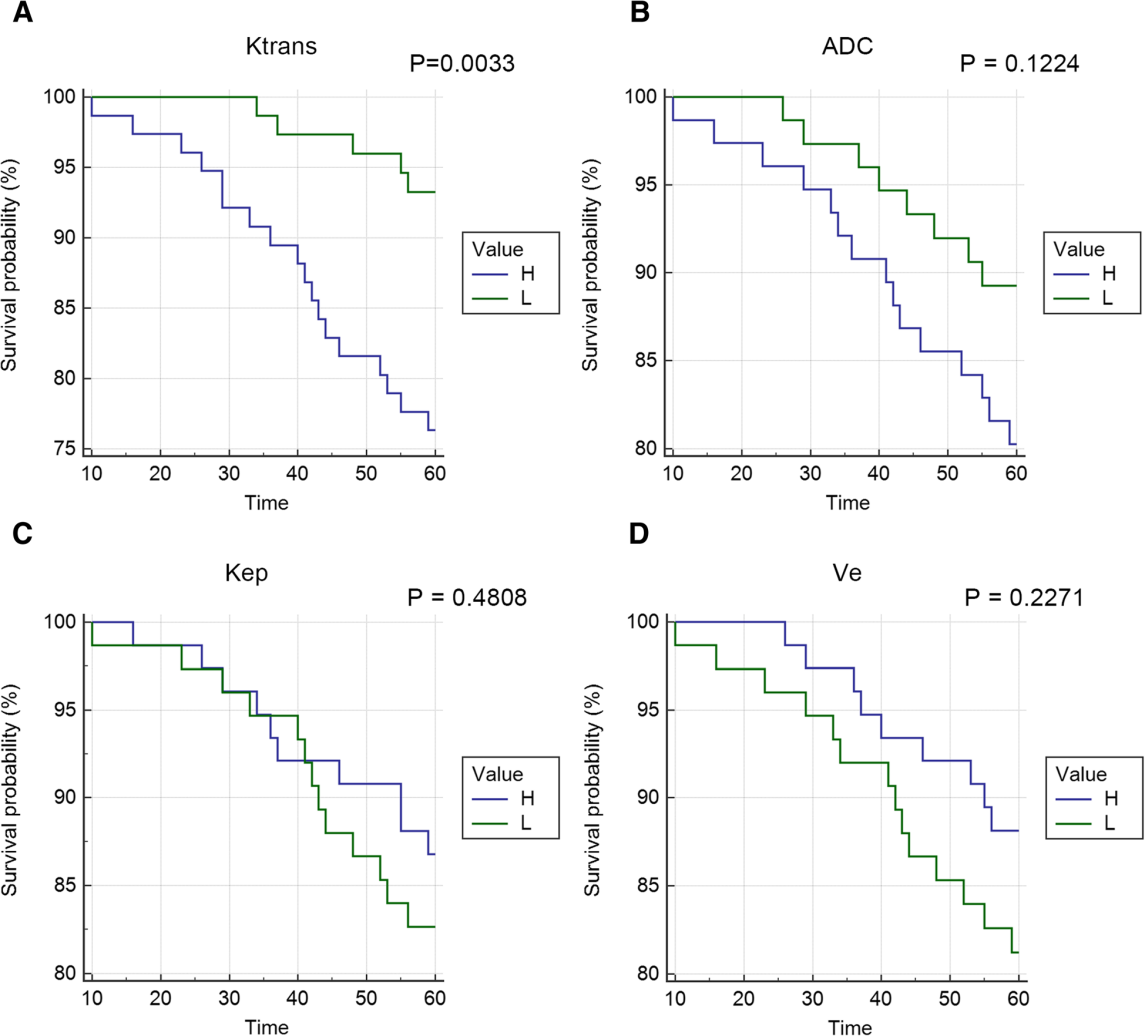
**a**–**d** Overall survival curves of patients for ADC values or DCE-MRI quantitative parameters

To further evaluate the risk of ADC values or DCE-MRI quantitative parameters to prognosis of breast cancer, Cox regression model was used (Table 5). The hazard ratio (HR) of ADC, Ktrans, Kep, and Ve values on survival was 5.26 (*P* = 0.093), 1.081 (*P* = 0.002), 1.006 (*P* = 0.941), and 0.883 (*P* = 0.926), respectively. However, Kaplan-Meier curve showed that only Ktrans value revealed significant effect on overall survival (Fig. 5).

**Table 5:** Hazard ratio (HR) of ADC values or DCE-MRI quantitative parameters on prognosis of breast cancer

Covariate	*b*	SE	*P*	HR	95% CI of HR
ADC	1.66	0.987	0.093	5.26	0.768 to 36.026
Ktrans	0.078	0.024	0.002	1.081	1.030 to 1.134
Kep	0.006	0.084	0.941	1.006	0.854 to 1.185
Ve	−0.125	1.337	0.926	0.883	0.065 to 11.960

## Discussion

**Fig. 6:**
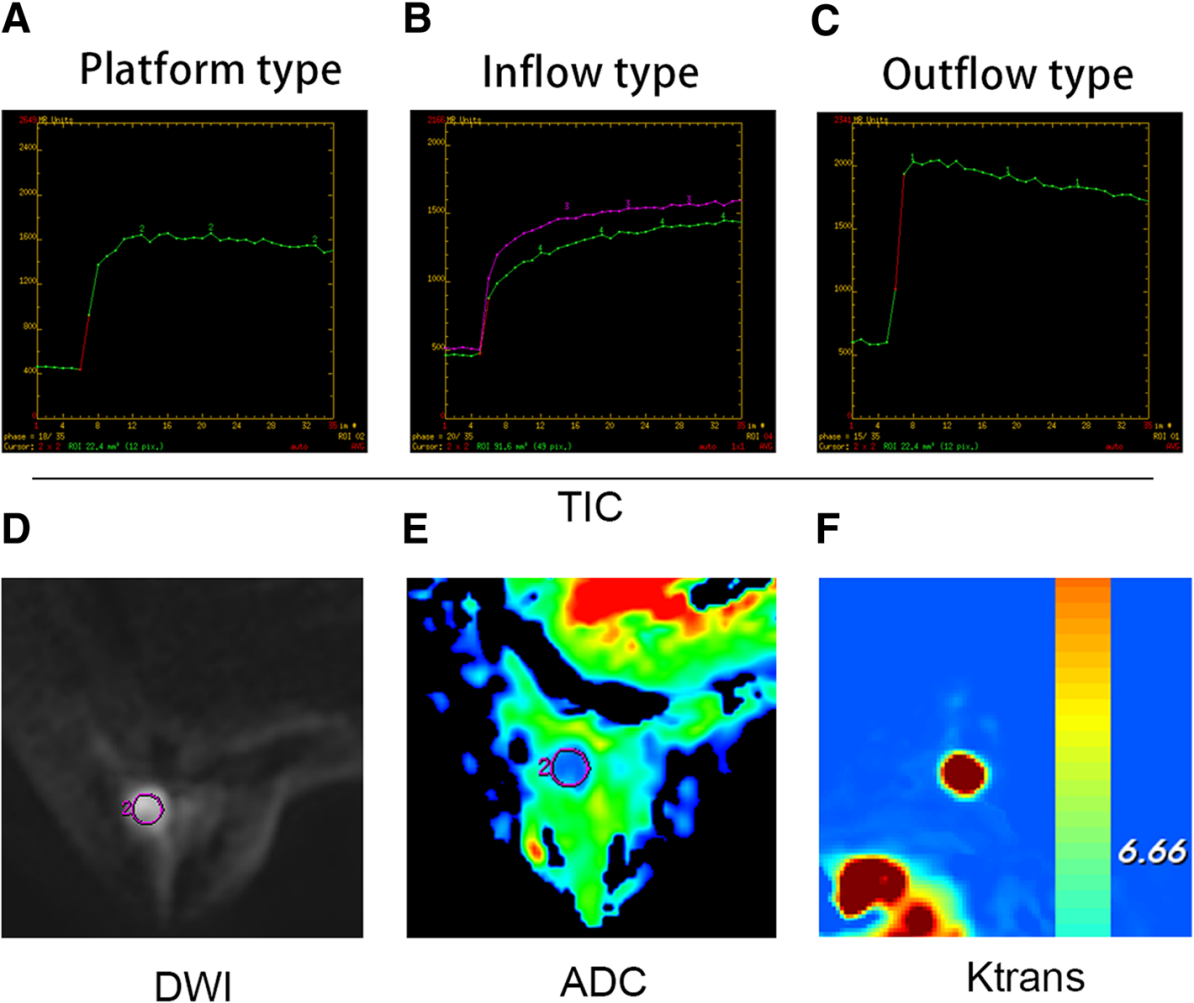
**a** Platform type TIC curve. **b** Inflow type TIC curve. **c** Outflow type TIC curve. **d** Diffusion-weighted imaging (DWI) map. **e** Apparent diffusion coefficient (ADC) image. **f** Ktrans image

Currently, breast MRI imaging not only shows the morphological characteristics of the lesion, but also provides hemodynamics, functional morphology, molecular imaging, and other aspects of the information, which greatly improve the accuracy of the diagnosis of breast disease and provide more comprehensive information for reasonable clinical treatment [17, 19, 20]. In addition to the conventional T1WI and T2WI sequences, the MRI sequences used in the diagnosis of breast cancer are mainly DCE-MRI, DWI, 1H-MRS and perfusion-weighted imaging (PWI), among which DCE-MRI and DWI are the most widely used and mature MRI techniques [21, 22]. Sample photos for reference are shown in Fig. 6.

Currently, the correlation between DCE-MRI quantitative parameters and prognosis factors of breast cancer, including enhanced morphology, tumor size, pathological grade, and lymph node status, is still in the preliminary stage, and the results of different studies are quite different [23, 24]. Koo et al. investigated the correlations between parameters (Ktrans, Kep, and Ve) of 70 IDCs and tumor size [23]. Their results revealed that mean values of Ktrans and Kep were higher in tumors with size > 2 cm than that with size ≦ 2 cm, respectively, whereas mean values of Ve were lower in tumors with size > 2 cm than that with size ≦ 2 cm. In consistent with this report, our study revealed that the mean values of Ktrans and Kep were apparently higher in tumors with size ≥ 2 cm than that with size < 2 cm, respectively. Our results also showed that significant differences were observed for the Ktrans values of subgroups of tumor size, but not for Kep and Ve values. However, Spearman correlation test showed a significant and positive correlation between Ktrans or Kep values and tumor size. In addition, DWI technology is the only imaging method to observe the microscopic movement of water molecules in living body [25–27]. It can reflect the spatial composition information of human tissue and the functional changes of water molecules of various tissues in the pathophysiological state [25–28]. We further found that though no significant differences were observed for the correlation between ADC values and tumor size, the ADC values between subgroups of tumor sizes were significantly different. These results suggested that Ktrans had the highest efficiency in evaluation of tumor size in this study. However, several studies reported that DWI shows strong potential as an adjunct MRI technique to reduce breast biopsies [29], demonstrating better performance than DCE-MRI [29–32]. Boulogianni et al. reported that combination with DCE improves the accuracy of cancer detection [30].

Histologic grade of tumor is a routine and important task for clinical diagnosis of IDC [33]. Recent research reported a positive correlation between ipsilateral whole-breast vascularity and tumor size, nuclear grade, and histologic grade, which manifested as higher Ktrans and Kep values [34–36]. Kim et al. studied 50 patients with different histologic grades of breast cancer lesions and showed that there was no significant difference in Ktrans values between higher and lower histologic grades [36]. However, our results revealed that only the Ktrans values had significant difference between subgroups of histologic grade. Thus, although there is no completely consistent view on the relationship between these parameters and histologic grade, these researches highly suggested that high histologic grade is clearly associated with higher Ktrans.

The presence of axillary lymph node metastasis is an important prognostic factor in patients with breast cancer, determining the followed management after surgery [37]. Our results also showed that Ktrans has significant positive correlation with LNM, while only Ve values exhibit significant difference between subgroups of LN, which is firstly reported in this study to date. Bahri et al. reported that LN(+) group has higher Kep than the LN(−) group [24]. The inconsistent results may be due to the relatively small sample size.

Breast cancer patients with ER- and PR-positive are sensitive to endocrine therapy, the survival rate is high, and the prognosis is relatively good [38]. When the ER and PR expression was negative, more contrast agent backflows from extravascular and extracellular space back to the tumor capillary cavity, indicating the capillary permeability is higher. Studies have shown that ER can reduce vascular endothelial growth factor (VEGF) levels and inhibit tumor angiogenesis [39]. Thus, compared with ER and PR positive expression, in tumor with ER and PR negative expression, the neovascularization is richer and the hemodynamic parameters may be higher [40]. Our study showed that ER and PR expression has negative correlation with ADC and Ktrans values, respectively. Hence, in consistent with previous researches, our results suggested that ADC and Ktrans values can reflect the information that the contrast agent diffuses from inner vessel into outer vessel. However, Ktrans value is a better indicator for the prediction of ER and PR expression compared to ADC value.

HER2, a product of a proto-oncogene, is a growth factor receptor with tyrosine kinase activity, which is rarely expressed in normal tissues [41]. Whether it is expressed or not is closely related to the occurrence and prognosis of breast cancer [42]. Our results showed that there was statistically negative correlation between HER2 and Kep values and positive correlation between HER2 and ADC values. However, some researches revealed that ADC values of IDC with HER2-positive are lower than those of IDC with HER2-negative because of increased cellularity [43]. These contradictory results may be due to the different values of *b* used in the determination of ADC [17]. Our results were highly consistent with the reports of Park et al., where *b* values of 0 and 1000 [17], the same with this studies, were used in the measurements of ADC values. Thus, we suggest that ADC, followed Kep, is the best predictor of HER2, which is closely related to the occurrence and prognosis of breast cancer.

Ki-67 is a nuclear antigen, which is associated with cell proliferation [44]. The higher the expression level of Ki-67 in tumor, the risk of recurrence is obviously increased [44]. In the current study, there were notably positive correlations between Ki-67 and Ktrans values. Consistent with our results, Kim et al. found that the Ktrans values in breast cancer with Ki-67 overexpression were significantly higher in patients with low expression of Ki-67 [38]. However, unlike Kim et al.’s study, there was no correlation between Kep value and Ki-67 in the present study. Thus, we suggested that the higher the Ktrans value is the tumor differentiation is worse, the degree of malignancy is higher, and possibility of tumor recurrence is greater.

In this study, higher Ktrans values are closely associated with higher rise of poor prognosis of breast cancer, while ADC, Kep, and Ve cannot predict prognosis of breast cancer. A similar result reported that ADC value was a good parameter in prediction of malignant breast tumors; however, ADC value was not a predictor of patient prognosis [45]. In addition, another study showed that compared with traditional DWI, diffusion kurtosis imaging (DKI) can better distinguish between benign and malignant breast lesions. Also, this article showed that Kep performed better than Ktrans [46]. However, our results suggested that Ktrans value is the best one in predicting prognosis of breast cancer. Therefore, further studies needs to be performed.

## Conclusions

We found that with higher values of Ktrans, ADC within IDC patients are associated with poor prognostic factors. Higher Kep values in IDC were closely positively correlated with tumor size and negatively correlated with PR and HER2. Ktrans value is helpful in predicting prognosis of breast cancer.
